# Curcumin and Papain-Loaded Liposomal Natural Latex Dressings with Phototherapy: A Synergistic Approach to Diabetic Wound Healing

**DOI:** 10.3390/ph18071067

**Published:** 2025-07-20

**Authors:** Franciéle M. Silva, Jaqueline R. Silva, Wellington Rodrigues, Breno A. S. M. Sousa, Thamis F. S. Gomes, Mario F. F. Rosa, Suélia S. R. F. Rosa, Marcella L. B. Carneiro

**Affiliations:** 1 Institute of Biological Sciences, University of Brasília, Brasília 73345-010, DF, Brazil; francielematos75@gmail.com (F.M.S.); rdasilvajaqueline@gmail.com (J.R.S.); 2 Faculty of Gama, University of Brasília, Brasília 73345-010, DF, Brazil; shipaniq@gmail.com (W.R.); brenomarinhos@gmail.com (B.A.S.M.S.); mariorosafleury@gmail.com (M.F.F.R.); 3 Laboratory of Bioactive Compounds and Nanobiotechnology (LCBNano), Campus Darcy Ribeiro, University of Brasilia, Brasilia 70910-900, DF, Brazil; thamisunb@gmail.com; 4 Meinig School of Biomedical Engineering, Master of Engineering Program, Cornell University, Ithaca, NY 14850, USA; sdf73@cornell.edu

**Keywords:** diabetes, wound dressing, wound healing, nanotechnology, latex, curcumin, papain, phototherapy

## Abstract

**Background:** Wound healing in diabetic individuals is a prolonged process, often complicated by infections and impaired tissue regeneration. Innovative strategies combining natural bioactive compounds are needed to enhance repair. **Methods:** This study reports the development and characterization of natural latex-based biomembranes (NLBs) incorporated with liposome-encapsulated curcumin and papain. The therapeutic efficacy of these composite dressings, in combination with red light-emitting diode (LED) phototherapy, was evaluated in a diabetic rat model. NLBs were produced by blending natural latex with multilamellar liposomes containing either curcumin, papain, or both. In vivo wound healing was assessed by applying the biomembranes to the dorsal lesions and administering red LED irradiation (650 ± 20 nm, 10 min every 48 h) over 11 days. **Results:** Fourier transform infrared spectroscopy (FTIR) confirmed that the integration of liposomes did not induce significant chemical alterations to the latex matrix. The treated diabetic rats exhibited enhanced wound contraction, with the curcumin and papain groups demonstrating up to 99% and 95% healing, respectively. Plasma fructosamine levels were significantly reduced (*p* < 0.05), indicating improved glycemic control. **Conclusions:** Combining NLBs with bioactive-loaded liposomes and phototherapy accelerated wound healing in diabetic rats. This multifunctional platform shows promise for the treatment of chronic wounds in diabetic patients.

## 1. Introduction

Diabetes mellitus (DM) is a chronic metabolic condition characterized by persistently elevated blood glucose levels, which contribute to neurological and vascular dysfunctions and impair tissue repair mechanisms. These alterations favor the development of chronic wounds, such as diabetic foot ulcers, which pose major clinical challenges and have a significant global economic impact [1,2,3]. Continuous hyperglycemia is associated with widespread organ damage, manifesting as both microvascular and macrovascular complications, thereby substantially increasing morbidity and mortality [4,5]. In addition, high glucose levels interfere with the activity of essential growth factors, hindering re-epithelialization and angiogenesis. Meanwhile, poor circulation, nutrient deficiencies, and chronic inflammation promote recurrent infections, which are commonly observed in diabetic ulcers [4,6].

Wound healing is a complex and dynamic process involving multiple cell types and biochemical pathways, from injury to complete regeneration. Under normal conditions, minor acute wounds heal within days to weeks without medical intervention. However, chronic wounds arise due to disruptions in the normal healing process, which is divided into three overlapping phases: hemostasis/inflammation, proliferation, and remodeling [7,8]. These phases must occur in an integrated, orderly, and timely manner to prevent chronic wound development and promote tissue restoration.

Traditional ulcer treatments, including activated charcoal and debridement, are often costly and ineffective. Consequently, there is a growing need for innovative approaches that leverage new materials or modifications of conventional ones to improve cost-effectiveness and therapeutic outcomes [8,9].

In this context, bioactive polymers have gained attention for their potential in wound healing. For instance, natural latex derived from the rubber tree *Hevea brasiliensis* is a bioactive polymer with excellent biocompatibility [10] and significant potential for tissue regeneration and other biomedical applications [11]. Studies have shown that latex promotes vascularization, angiogenesis, and accelerates wound healing in animal and human models [12,13,14,15,16,17]. Additionally, a protein fraction of latex has been shown to enhance antioxidant activity and reduce the release of pro-inflammatory cytokines, further supporting its therapeutic potential [18].

Natural compounds are widely used in wound healing due to their phytochemical constituents, which can act in tissue regeneration due to anti-inflammatory, antioxidant, and antimicrobial properties [19,20]. Among these, curcumin, a bioactive compound with anti-inflammatory, anti-infective, anticancer, immunomodulatory, antioxidant, and wound healing activities, holds promise for treating chronic wounds. However, its hydrophobic nature limits its bioavailability, posing challenges for its pharmaceutical development [21].

Similarly, papain, a proteolytic enzyme extracted from papaya latex, contains essential nutrients and bioactive compounds, such as antioxidants, vitamins, and minerals. Papain exhibits anti-inflammatory, antibacterial, and antioxidant properties, contributing to its wound healing effects. Its enzymatic debridement activity and role in collagen synthesis (via vitamin C-mediated conversion of proline to hydroxyproline) make it particularly valuable for tissue repair [22,23,24].

Recent advancements in nanotechnology have also revolutionized wound treatment. This field offers innovative solutions, including the use of advanced nanomaterials as dermal substitutes or drug delivery systems for bioactive molecules, thereby enhancing the healing process [19]. Liposomes as a class of nanomaterials have emerged as promising therapeutic vehicles due to their biocompatibility, biodegradability, low toxicity, and immunogenicity. Their amphipathic nature allows for the encapsulation of both hydrophobic and hydrophilic molecules, enabling sustained release of bioactive compounds [25]. For example, liposomes can enhance the solubility and bioavailability of curcumin, facilitating its incorporation into an NLB for wound healing applications, as explored in this study. The use of liposomes is likely to be successful since the protective lipid bilayer increases stability and protects the drug from degradation, leading to improved bioavailability [26,27].

Photobiomodulation is a non-invasive technique that employs light at specific wavelengths, such as red or near-infrared, to stimulate biological responses in tissues. This type of irradiation can enhance ATP production, modulate oxidative stress, induce the release of growth factors, and stimulate cell proliferation [28,29]. These properties make photobiomodulation particularly effective in tissue regeneration and have led to its widespread use in clinical contexts such as the healing of chronic wounds, diabetic ulcers, bone repair, and musculoskeletal injuries [30,31]. In addition to accelerating wound healing, this technique can reduce inflammation, modulate the local immune response, and alleviate pain, thereby promoting more efficient recovery with fewer adverse effects [32,33].

A therapeutic system called Rapha^®^ has been developed at the University of Brasília, used for application in inflammatory processes and tissue regeneration. The Rapha^®^ system consists of the application of red LED phototherapy and a biomembrane, based on natural latex extracted from the rubber tree *Hevea brasiliensis* (NLB), in the ulcer [12,34,35,36,37]. In this study, we developed natural NLBs incorporating liposomes loaded with curcumin and papain, aiming to enhance wound healing in diabetic rats. These biomembranes, combined with LED phototherapy, offer a promising approach for tissue regeneration, leveraging the biological properties of curcumin and papain to boost the healing process.

## 2. Results

### 2.1. Characterization of Liposomes

The liposomal formulations presented a characteristic multilamellar profile. The dual-loaded liposomes (containing both curcumin and papain) exhibited an average vesicle size of 905.5 ± 122.3 nm, a polydispersity index (PDI) of 0.764, and a zeta potential of −40.7 ± 2.22 mV. These values reflect the typical physicochemical characteristics of the multilamellar liposomes used in the study. Due to limitations in sample availability and instrument access during the experimental phase, individual characterization of the curcumin-only and papain-only liposomes was not performed. Therefore, comparative data among all liposome types were not generated.

**Figure 1:**
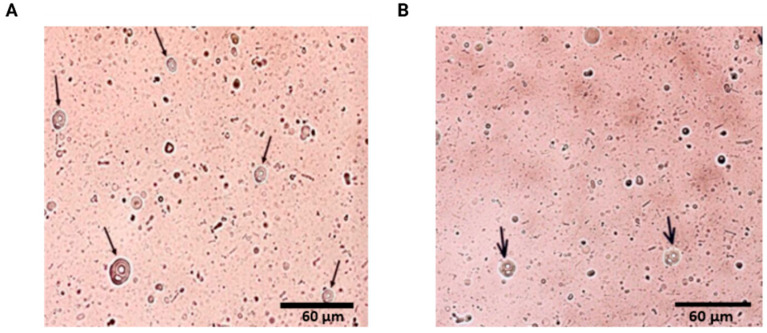
Photomicrographs of multilamellar liposomes (indicated by arrows). Following the preparation of the liposomes, an aliquot was diluted in ultrapure water (1:100), placed on a slide, and covered with a coverslip. The samples were then observed under the EVOS FL Auto Cell Imaging System microscope (Thermo Fisher Scientific, Waltham, MA, USA) using a 40× magnification objective. (**A**) Multilamellar liposomes containing curcumin and papain and (**B**) multilamellar empty liposomes.

Microscopic analyses confirmed the structural integrity and morphology of the vesicles. As shown in Figure 1, the dual-loaded liposomes (Figure 1A) displayed a rounded shape and well-defined lamellae, consistent with a multilamellar configuration. The empty liposomes (Figure 1B) showed similar morphological features, indicating that the incorporation of curcumin and papain did not significantly alter the visual structure of the vesicles. No significant precipitation or phase separation was observed following preparation.

### 2.2. Characterization of Biomembranes by Fourier Transform Infrared Spectroscopy (FTIR)

**Figure 2:**
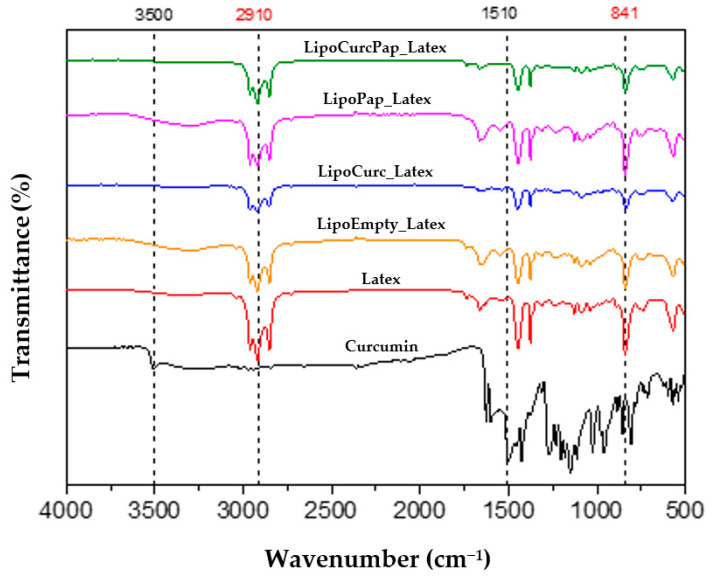
Infrared spectra of curcumin (black), latex biomembranes (Latex, in red), empty liposomes in latex biomembranes (LipoEmpty_Latex, in orange), curcumin-loaded liposomes in latex biomembranes (LipoCurc_Latex in blue), papain-loaded liposomes in latex biomembranes (LipoPap_Latex, in pink), and curcumin plus papain-loaded liposomes in latex biomembranes (LipoCurcPap_Latex, in green).

Figure 2 presents the FTIR spectra of latex, curcumin, papain, and all liposomal preparations. The latex bands exhibited characteristic bands at 837 cm^−1^, corresponding to =CH out-of-plane vibrations, which are the most significant absorptions for identifying natural latex as they characterize the R_2_C=CHR functional group (cis-1,4-polyisoprene). We observed additional bands at 567 cm^−1^ (C-C deformation), 1240 cm^−1^ (asymmetric stretching), between 2852 cm^−1^ and 2925 cm^−1^ (symmetric stretching vibrations of CH_2_), around 3040 cm^−1^ (CH stretching), between 2963 cm^−1^ and 2845 cm^−1^, 1658 cm^−1^ (C-H_3_ and CH_2_ stretching), 1445 cm^−1^ (C=C stretching), and 1374 cm^−1^ (CH_2_ and CH_3_ deformation).

We identified bands in the FTIR spectra of curcumin that displayed characteristic bands associated with its chemical structure, including a broad region from 3300 cm^−1^ to 3410 cm^−1^ (phenolic O-H stretching vibration), bands at 1640 cm^−1^, 1645 cm^−1^, and 1647 cm^−1^ (carbonyl C=O group), bands between 1345 cm^−1^ and 1474 cm^−1^ (C=C vibrations of the aromatic ring and CO bond), regions from 1020 cm^−1^ to 1155 cm^−1^ (C-O ether group stretching), and bands between 700 cm^−1^ and 900 cm^−1^ (C-H alkenes groups). We observed no significant differences in the FTIR spectra of the NLB after the incorporation of liposomes, indicating that the addition of these vesicles did not alter the chemical or structural properties of the latex.

The results of FTIR analyses of NLBs containing liposomes demonstrate that the characteristic functional groups of each sample were observed. No additional absorption bands, other than those already present in natural latex, curcumin, and papain, were identified.

### 2.3. Production of Natural Latex Biomembranes (NLBs)

**Figure 3:**
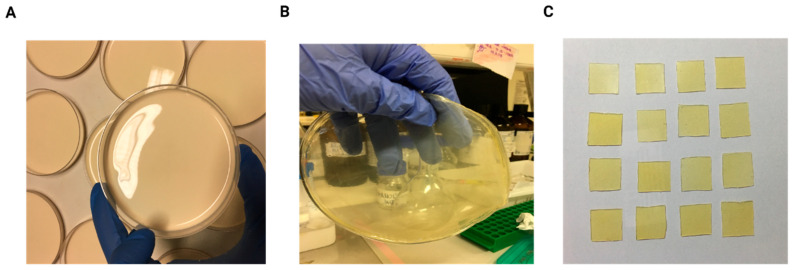
Preparation of natural latex biomembranes. (**A**) Liquid latex is mixed with ultrapure water in a 1:1 ratio and poured into plastic Petri dishes. (**B**) The mixture is then oven-dried at 40 °C for 24 h to form solid biomembranes. (**C**) After drying, the biomembranes are cut into 2 × 2 cm squares.

NLBs were produced by mixing liquid latex (DU LATEX Indústria Química Ltd., São Paulo, Brazil) with ultrapure water in a 1:1 volume ratio. The resulting solution was poured into plastic Petri dishes measuring 90 × 15 mm (Figure 3A) and dried in an oven at 40 °C for 24 h, allowing biomembrane formation (Figure 3B). After polymerization, the NLBs were cut into 2 × 2 cm squares for subsequent experimental use (Figure 3C). The final NLB format was selected based on its physicochemical properties, such as transparency, thickness, and malleability, which facilitated adhesion to the animals’ skin in in vivo experiments.

### 2.4. In Vivo Tests

#### Clinical Aspects

**Figure 4:**
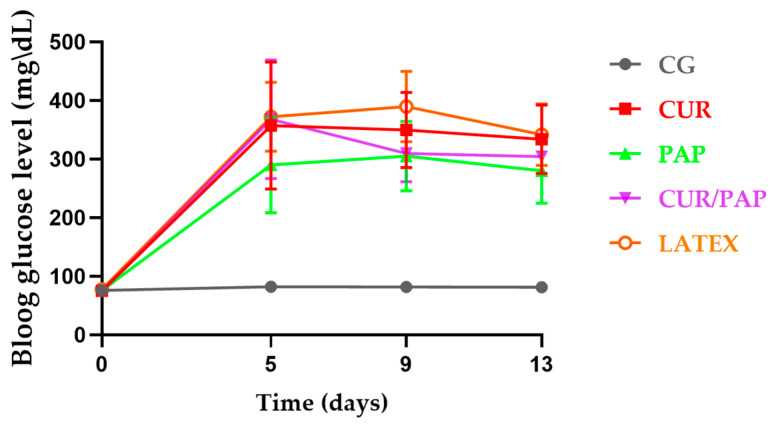
Blood glucose levels of rats throughout the experiment. Temporal changes (days) in blood glucose levels (mg/dL). There were no significant differences between the groups.

Figure 4 illustrates the changes in fasting blood glucose levels in rats. On day 0, the animals exhibited blood glucose levels within the normal range. Throughout the experiment, blood glucose levels were monitored on days 0, 5, 9, and 13. It is important to note that day 0 corresponds to ten days after the alloxan injection, when the experimental protocol began, and hyperglycemia was already established. Ten days after the alloxan injection, the animals developed marked hyperglycemia, with blood glucose levels exceeding 250 mg/dL, compared to pre-diabetic levels of approximately 88 mg/dL.

**Figure 5:**
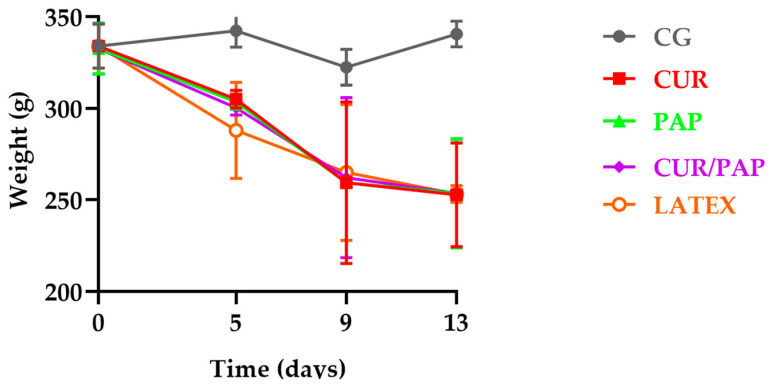
Body weight during the experimental period. On day zero, the animals were non-diabetic. However, from day 5 to day 13, the animals developed diabetes. Data are expressed as mean ± standard error of the mean (SEM) for each group (*n* = 5). Significant differences were observed when comparing body weight on day zero with that on day 13.

We observed loss of body weight in all diabetic groups (*n* = 5), a well-known indicator of diabetes, as shown in Figure 5. Additionally, clinical signs such as polyuria and polydipsia were noted in the animals. No deaths or adverse events were observed throughout the entire experimental period.

During the experiment, the animals exhibited clinical signs of diabetes, including weight loss, as illustrated in Figure 5. Polyuria was evidenced by the need to change the cage shavings daily due to excessive wetness caused by increased urine output.

### 2.5. Biochemical Assessment: Plasma Fructosamine

The induction of diabetes resulted in elevated blood glucose levels, increased plasma fructosamine concentrations, and the presence of glycosuria. In this study, we noted that alloxan monohydrate increased plasma fructosamine levels in the rats (Table 1; *p* < 0.05). Statistical analysis revealed that the control group had significantly lower fructosamine levels compared to the treatment groups, which consisted of diabetic animals. The GC group showed significantly lower fructosamine levels compared to the G1 (*p* = 0.001), G3 (*p* = 0.013), and G4 (*p* = 0.003) groups, while the difference compared to the G2 group was not statistically significant (*p* = 0.053). Similarly, fructosamine levels in the GC-ii group were significantly lower than in all diabetic experimental groups subjected to treatment (*p* < 0.001).

### 2.6. Evaluation of Wound Healing

**Figure 6:**
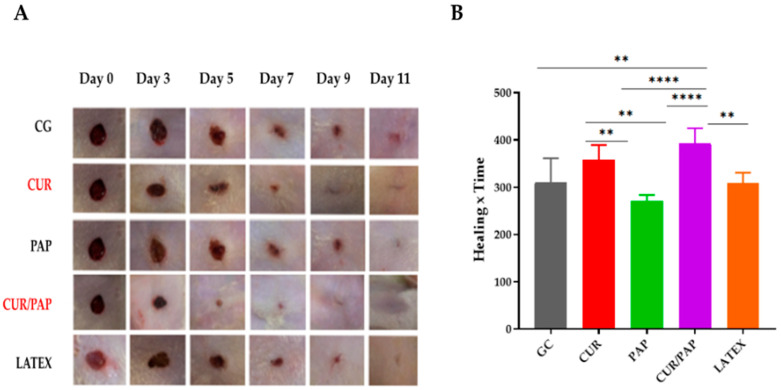
Evolution of wound healing images from in vivo experiments. The CUR and CUR/PAP groups, highlighted in red, demonstrated superior wound healing outcomes compared to the other groups. (**A**) Wound healing in diabetic Wistar rats: CG—untreated control group; CUR—group treated with latex biomembrane containing curcumin-loaded liposomes + LED phototherapy; PAP—group treated with latex biomembrane containing papain-loaded liposomes + LED phototherapy; NLB + CUR and PAP liposomes + LED and LATEX—group treated with latex biomembrane containing curcumin- and papain-loaded liposomes + LED phototherapy. (**B**) Healing curve (AUC): The area under the curve was calculated to analyze the overall healing effect throughout the entire experimental period rather than a specific time point. Data *(n* = 5) are expressed as ± standard error of the means (SEM) with a 95% confidence interval (** *p* < 0.01, **** *p* < 0.0001). Statistical analysis was performed using one-way ANOVA followed by Tukey’s multiple comparisons test.

Compared to the control group, the treated groups demonstrated a significantly faster wound healing process in rats (Figure 6A). Figure 6B illustrates the AUC of wound healing over time. The AUC values, derived from the healing area, were plotted to generate a curve representing the progression of wound healing throughout the experimental period. The results are expressed as the integrated area under the healing curve.

The use of NLB enriched with curcumin and papain liposomes, combined with LED phototherapy, significantly affected healing in diabetic rats. The results revealed that the groups treated with these biomembranes could show a significant contraction of the lesion surface, with the curcumin-treated group achieving an average contraction of 99% (*p* < 0.01) and the papain-treated group a contraction of 95% (*p* < 0.05). This treatment was statistically superior to the control group, suggesting a synergistic potential between the incorporated bioactive compounds and phototherapy. In addition, analysis of the AUC revealed that the combined therapy advanced healing and produced a more intense response over the experimental period (*p* < 0.0001 compared to all other groups).

Another finding that helped to elucidate the process was the marked reduction in plasma levels of fructosamine in the treated groups, showing more effective glycemic control during healing. This finding validates the hypothesis that latex biomembranes with liposomes can exert a local therapeutic effect on the wound and systemically contribute to the metabolic balance of diabetic animals. This dual effect, on skin healing and blood glucose control, demonstrates the potential of these biomembranes as a promising, multifunctional approach to treating chronic wounds in diabetic patients.

## 3. Discussion

This study investigated the therapeutic potential of a natural latex biomembrane (NLB) incorporated with liposomes loaded with curcumin and/or papain, combined with red LED photobiomodulation. Our findings represent a relevant advance in the field of wound care, demonstrating that this multifunctional dressing enhances tissue repair in a diabetic model through a synergistic action of bioactive compounds and light therapy.

The combination of curcumin and papain in the NLB improved wound contraction, with values of 99% and 95%, respectively, compared to NLB and LED alone. The therapeutic enhancement observed suggests that bioactive agents and LED act synergistically to stimulate tissue regeneration mechanisms, corroborating previous studies [34,38].

The FTIR spectra obtained for the NLBs showed characteristic bands matching those reported in the literature, confirming the successful incorporation of curcumin and papain into the NLB matrix. For instance, our findings align with those reported by Gemeinder et al. [39], displaying characteristic bands of the latex biomembrane functional groups at 2910 cm^−1^ and 841 cm^−1^. For curcumin, the spectra revealed stretching vibrations at 3500 cm^−1^ (O-H groups) and between 1500 cm^−1^ and 500 cm^−1^ (C=O mixed vibrations), consistent with the findings of Ismail and co-workers [40].

Liposomes were selected as delivery systems due to their well-known biocompatibility, biodegradability, and ability to encapsulate both hydrophilic and hydrophobic agents. These features have significantly enhanced the versatility of NLBs in drug delivery systems since they can deliver their cargo to the NLBs through protein associations [41]. The multilamellar vesicles used in this study were prepared with a 7:3 phospholipid-to-cholesterol ratio, which supports structural stability and efficient co-encapsulation [25,42]. Despite elevated PDI values [43], wound healing was not compromised, and liposomes effectively delivered curcumin and papain even with a negative surface charge [44,45]. This performance is consistent with a sustained release profile that enhances therapeutic activity [44].

While positively charged nanoparticles generally exhibit greater cellular permeability [44,45], we observed that, despite presenting a negative surface charge, the liposomes did not hinder the delivery of curcumin and papain, as evidenced by the enhanced healing observed in the experimental groups treated with these phytotherapeutics. These nanoparticle characteristics suggest a sustained drug release profile [44], which may improve the delivery of phytocompounds such as curcumin and promote wound healing [45].

Zhai and co-workers (2024) demonstrated that hydrogels can be functionalized with zinc oxide nanoparticles, enhanced collagen deposition and reduced inflammation and oxidative stress in diabetic wounds [46]. Similarly, chitosan-coated zinc oxide nanocomposites exhibited antibacterial activity and improved collagen repair, reinforcing the potential of nanotechnology in tissue regeneration [47].

Clinically, our group has previously reported that curcumin-loaded NLBs promoted an average ulcer shrinkage rate of 89.9% in a pilot study involving diabetic patients [34]. The therapy outperformed the gold standard in the Brazilian Unified Health System, demonstrating substantial wound reduction. These observations are consistent with our current findings and with other studies that highlight the adhesiveness, tenacity, and healing potential of NLBs [17,48]. In this work, we have demonstrated, for the first time, the development of an NLB containing bioactive compounds. These findings support the efficacy of combined approaches—such as the treatments proposed in the present study—for applications in wound healing, including chronic and infected wounds.

Curcumin’s therapeutic efficacy is well documented for wound treatment, especially due to its anti-inflammatory properties, including the inactivation of reactive oxygen species and the reduction in lipid peroxidation. Additionally, this natural compound may enhance cell proliferation, stimulate collagen synthesis and maturation, and promote extracellular matrix biosynthesis [49,50]. Topical administration has shown superior results over oral intake in wound repair [51].

Furthermore, Sidhu et al. highlighted the efficacy of curcumin treatments, whether applied topically or orally, in promoting wound healing in diabetic rats and genetically diabetic mice [52]. However, due to its poor solubility, encapsulation in liposomes is necessary to enhance bioavailability and stability [23]. The amphipathic nature of liposomes allows for sustained release of curcumin and papain, optimizing wound healing outcomes [53,54].

The efficacy of liposomal curcumin has been corroborated by studies using hydrogel platforms for diabetic wound healing. Liu and co-workers (2018) demonstrated improved wound closure, flexibility, and antimicrobial protection with curcumin-loaded gelatin microsphere hydrogels [55]. Other investigations have also shown that curcumin-incorporated hydrogels stimulate collagen proliferation and minimize scarring [56,57].

In this study, we used liposomes to encapsulate curcumin within the NLB matrix. This strategy effectively addresses the inherent limitations of curcumin, such as poor solubility and low bioavailability, while enabling its therapeutic application [58]. The feasibility of using lipid-based nanocarriers for bioactive delivery is supported by prior work. For example, Sapkota et al. [59] developed papain-loaded liposomes and transferosomes, demonstrating stable vesicle formation and preserved enzymatic activity through dynamic light scattering. Their formulation proved effective in managing hypertrophic scar, reinforcing the therapeutic potential of proteolytic enzymes in lipid-based systems.

Likewise, several studies have reported the successful development of liposomal and other lipid-based nanocarrier systems for curcumin, showing improvements in physicochemical stability, bioavailability, and therapeutic efficacy in wound healing. Liu et al. [55], for instance, designed curcumin-loaded thermosensitive hydrogels incorporating nanoparticles, which enhanced collagen deposition, reduced inflammation, and accelerated wound closure in diabetic mice. Similarly, Shefa et al. [56] incorporated curcumin into oxidized cellulose nanofiber–polyvinyl alcohol hydrogels, achieving sustained release and significant tissue regeneration in vivo. Although our study did not include full characterization of papain-only liposomes, the literature provides a robust foundation that validates the structural feasibility and therapeutic relevance of this approach.

Moreover, curcumin-based nanoformulations have demonstrated significant benefits in treating infected burns and promoting wound healing, as they enhance curcumin’s bioavailability and enable sustained drug release [50,51,60]. Here, we present a novel product solution for diabetic wound dressings that combines nanotechnology with tissue-forming properties. The most promising results were achieved with a latex-based liposome loaded with curcumin, which aligns with findings reported in the literature and reported by Gomes et al. al (2025) in their pilot clinical study [34]. Papain, a proteolytic enzyme from *Carica papaya*, has shown promising results in wound healing due to its bactericidal, bacteriostatic, and anti-inflammatory properties. Moreover, papain promotes the debridement of devitalized and necrotic tissue, accelerating the healing process. It promotes enzymatic debridement, stimulates cytokine production, and facilitates wound edge approximation [61]. In our study, papain-loaded liposomes significantly improved wound contraction, consistent with previous findings using *Carica papaya* extracts [62].

LED phototherapy is supported by evidence demonstrating its role in reducing inflammation, stimulating fibroblast proliferation and angiogenesis, enhancing granulation tissue formation, and increasing collagen synthesis [63,64]. Red light at 650 nm has been widely validated as an effective wavelength for enhancing wound healing. Its optimal tissue penetration enables stimulation of key biological processes such as fibroblast proliferation, angiogenesis, and collagen synthesis, without causing thermal damage. Mechanistically, 650 nm activates cytochrome c oxidase in mitochondria, enhancing ATP production and cellular metabolism without inducing thermal damage [65]. These effects, together with the established safety profile of LED in this context, strongly support the selection of 650 nm in the present study and demonstrate how phototherapy contributes to improving healing outcomes.

The therapeutic synergy observed in this study likely arises from the complementary mechanisms of curcumin, papain, and red LED photobiomodulation. Curcumin exerts potent anti-inflammatory and antioxidant effects by downregulating NF-κB, COX-2, and other inflammatory mediators, as well as by scavenging reactive oxygen species (ROS) and modulating extracellular matrix production [66]. These effects contribute to the restoration of redox balance and attenuation of chronic inflammation in the wound microenvironment. Papain, in turn, enhances granulation of tissue formation, angiogenesis, and collagen remodeling [67]. Red LED light further potentiates healing through mitochondrial activation and cellular stimulation [65]. Together, these mechanisms support enhanced outcomes observed.

The present study did not include control groups comprising NLBs incorporated with curcumin-loaded liposomes, papain-loaded liposomes, or their combination in the absence of LED photobiomodulation. This experimental decision was based on the primary objective of evaluating the therapeutic potential of a combined strategy, integrating bioactive liposomal formulations with red light irradiation. The wound healing efficacy of curcumin and papain has been extensively documented in the literature, with curcumin demonstrating anti-inflammatory, antioxidant, and proliferative effects [52,68], and papain exhibiting notable proteolytic and debriding activity conducive to tissue regeneration [59,69,70]. Similarly, red light photobiomodulation (approximately 650 nm) is well established as a non-invasive modality that enhances wound healing by promoting cellular metabolism, angiogenesis, and modulation of inflammatory mediators [71].

Interestingly, the reduction in fructosamine levels in treated animals suggests additional metabolic benefits. Curcumin and papain have demonstrated anti-glycation and antioxidant properties that may contribute to local wound microenvironment modulation and indirectly influence systemic metabolic markers [52,66]. Further studies will help elucidate whether this effect is primarily local or involves systemic absorption.

Overall, this study presents a promising strategy that integrates natural biomaterials, nanotechnology, and photobiostimulation to improve wound care. The multifunctional NLB platform incorporating liposomal curcumin and papain, combined with LED therapy, demonstrated superior healing in a diabetic model and reinforces its potential for translational applications.

### Study Limitations and Perspectives

While the alloxan-induced diabetic rat model provides a well-established platform for investigating impaired wound healing, it does not fully replicate the complexity of chronic wounds in human diabetic patients, particularly concerning microbial biofilms, comorbidities, and persistent inflammation. Nevertheless, in vivo testing remains essential in preclinical research to evaluate safety and therapeutic potential before clinical application. Although our model enabled controlled assessment of treatment efficacy, future studies should explore more clinically relevant systems, such as organ-on-a-chip platforms and computational models, which better simulate dynamic physiological conditions and improve translational predictability.

Importantly, the natural latex-based wound dressing used in this study has previously demonstrated safety and efficacy in human subjects [72,73]. This work focused on integrating bioactive liposomes—loaded with curcumin and papain—into an already validated clinical platform. As such, these preclinical findings represent a critical step toward advancing the formulation for future clinical trials [74,75,76].

Several methodological limitations must be acknowledged. Although Fourier transform infrared spectroscopy confirmed compound incorporation, additional structural (SEM) and thermal (DSC) analyses were not conducted. The liposomes exhibited high polydispersity index (PDI) values, suggesting heterogeneous particle size distributions; however, this did not impair their therapeutic performance. Likewise, despite a negative surface charge, the liposomes effectively delivered curcumin and papain, possibly due to a sustained release profile. These parameters should be further optimized and standardized to improve reproducibility.

Another relevant limitation relates to the physicochemical characterization of liposomal formulations. While particle size, PDI, and zeta potential were evaluated for the dual-loaded liposomes containing curcumin and papain, a complete comparative analysis of the individual formulations—empty, curcumin-loaded, and papain-loaded liposomes—was not performed. Although all formulations were prepared and incorporated into the biomembranes, assessing the specific impact of single-agent encapsulation on colloidal behavior was beyond the scope of this study. As drug loading can influence vesicle size, surface charge, and stability, future studies should conduct comprehensive physicochemical and morphological characterization of each formulation to enhance mechanistic insight and support formulation refinement.

The absence of stability data for the liposomal formulations under various storage conditions is also a limitation. Although the main goal was to assess the wound healing efficacy of the liposome-loaded NLBs, the long-term stability of the formulations remains to be established. According to ICH Q1A(R2) guidelines, stability studies are essential for determining shelf-life, defining storage conditions, and ensuring the robustness and reproducibility of pharmaceutical products. Future work should include both real-time and accelerated stability testing to support the regulatory advancement of this therapeutic platform.

Additionally, the LED parameters used in this study were based on device specifications and standard preclinical guidelines. However, in vivo measurements of light penetration and absorption were not conducted. Since variables such as skin pigmentation, vascularization, and anatomical location may influence light distribution, these factors should be investigated in future studies to ensure consistency across diverse clinical scenarios.

Regarding safety, no adverse effects were observed in animals treated with papain-loaded dressings. Nevertheless, papain is a proteolytic enzyme that may cause hypersensitivity reactions, particularly with occupational exposure or ingestion. While our formulation involves topical application within a natural latex matrix, the possibility of systemic absorption through compromised skin cannot be excluded. Thus, future studies should include toxicological and immunological assessments to evaluate sensitization potential, systemic exposure, and bioavailability. Establishing the immunological safety of repeated topical use will be critical for regulatory approval and clinical adoption.

Regulatory translation of latex-based biomaterials also requires compliance with safety standards, long-term biocompatibility studies, and the development of scalable, standardized manufacturing protocols that preserve bioactivity. Dose–response analyses, multicenter clinical trials, and cost-effectiveness studies will be essential for integration into healthcare systems.

Finally, the absence of specific control groups—such as NLBs containing bioactive liposomes without LED—limits the ability to distinguish additive from synergistic effects among the therapeutic components. This limitation has been addressed in the Discussion and considered in the interpretation of the results. Future studies should include additional comparative groups to clarify the individual and combined contributions of curcumin, papain, and phototherapy. Such efforts will be key to validating and optimizing this multimodal strategy for clinical translation.

Collaborative efforts across dermatology, endocrinology, materials science, and surgical specialties will also be fundamental to confirm therapeutic efficacy and define appropriate clinical indications. While our results support a promising integrative approach involving biomaterials, nanotechnology, and photobiomodulation, a multidisciplinary translational strategy will be essential to ensure safe, effective, and patient-centered application in clinical settings.

## 4. Materials and Methods

### 4.1. Preparation of Liposomes

Multilamellar liposomes were obtained using the solvent evaporation and thin-film lipid hydration method, as described by Mohammed et al. [77]. The process began with the dissolution of a lipid mixture composed of phospholipids [L-α-phosphatidylcholine from soybean (≥99% purity) was provided by Avanti Polar Lipids (Alabaster, AL, USA; product no. 840051P) and cholesterol (≥99% purity, Sigma-Aldrich, St. Louis, MO, USA; product no. C8667)], in a molar ratio of 7:3, in a solution of organic solvents (chloroform and methanol in a 3:1, *v/v* ratio), a proportion widely recognized in the literature for its suitability in topical and transdermal formulations [25,42].

After complete solubilization of the lipids, a dry lipid film was formed by evaporation of the solvents using a rotary evaporator (Rotavapor^®^ R-100, BUCHI Labortechnik AG, Flawil, Switzerland; manufactured by Büchi Operations India Pvt Ltd, Surat, India) for 2 h at 40 °C. This lipid film was then hydrated with a phosphate-buffered saline solution (PBS), pH 7.4, under vigorous magnetic stirring, resulting in the formation of liposomes.

**Figure 7:**
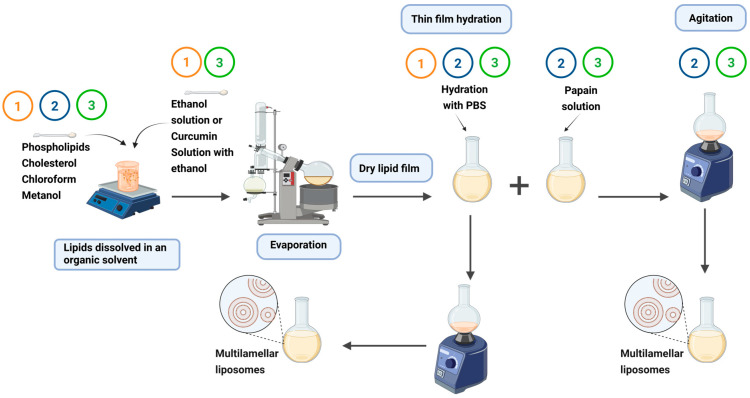
Production steps of three multilamellar liposome formulations: (1) liposomes containing curcumin prepared by adding curcumin (1 mg/mL in ethanol) to the lipid mixture before film formation, followed by hydration with PBS; (2) liposomes containing papain obtained by hydrating the lipid film with a papain solution (2.5 mg/mL in PBS); and (3) liposomes containing both curcumin and papain, produced by adding curcumin to the lipid phase, followed by hydration of the film with the papain solution. Created in BioRender. Silva, F. (2025) https://biorender.com/zievczv (accessed on 16 July 2025).

These liposomes are presented in Figure 7, illustrating the formation of liposomes from film hydration. Three types of multilamellar liposomes were prepared: one containing only curcumin, one only papain, and a third with both molecules in the same preparation. For the liposomes containing curcumin (HPLC grade, ≥94% purity; Sigma-Aldrich; product no. C1386), a solution of curcumin in ethanol (1 mg/mL) was included in the lipid mixture (1). In the liposomes loaded with papain (lyophilized papain from *Carica papaya*, ≥10 U/mg protein, 2× crystallized, enzyme grade; Sigma-Aldrich; product no. P4762), the lipid film was hydrated with a solution of papain (2.5 mg/mL) in PBS (2). Liposomes containing both molecules were obtained by combining the procedures described for curcumin and papain (3).

### 4.2. Characterization of Liposomes

After preparation, the multilamellar liposomes were initially evaluated for phase separation, curcumin (CUR) or papain (PAP) precipitation, and creaming formation under centrifugation at 1500 g (kasvi centrifuge K14-4000PRF) for 10 min at 10 °C. The mean hydrodynamic diameter and zeta potential were measured with a ZetaSizer^®^ (Nano ZS90, Malvern, UK) using dynamic light scattering (DLS) and electrophoretic mobility, respectively (90° angle). Size distribution and lamellarity were further assessed by inverted optical microscopy (EVOS™ M7000, Thermo Fisher Scientific, Waltham, MA, USA).

### 4.3. Production of Natural Latex Biomembrane (NLB)

Liposomal suspensions containing curcumin (5 mg/mL) and papain (2.5 mg/mL) were mixed at a 1:1 ratio with a natural latex solution. Ten milliliters of this mixture was poured into sterile polystyrene plates measuring 90 × 15 mm (13.5 cm^2^) and dried at 40 °C for 24 h. The resulting biomembranes were cut into 2 cm × 2 cm sections (4 cm^2^). Based on the surface area ratio and incorporated liposomal volume, each dressing contained approximately 7.4 mg of curcumin and 3.7 mg of papain, ensuring consistency in the dose of bioactive compounds delivered per treatment.

### 4.4. Preparation of Latex Biomembrane Containing Multilamellar Liposomes

The preparation of NLBs containing liposomes was carried out by adding curcumin- or papain-loaded liposomes to the latex solution at a ratio of 1:1. Ten milliliters (10 mL) of this mixture was placed in polypropylene Petri dishes and maintained in an oven at 40 °C for 24 h to produce the liposome-containing biomembranes.

### 4.5. Sterilization and Packaging of NLB

The prepared NLBs were sterilized by ultraviolet (UV) irradiation in a unidirectional laminar flow hood (CFLV 12-Veco, Veco Scientific Equipment, Campinas, São Paulo, Brazil) for 30 min on each side, totaling 60 min. After sterilization, each biomembrane was individually wrapped in 90 mm × 160 mm self-sealing surgical paper and stored until use.

### 4.6. Fourier Transform Infrared Spectroscopy (FTIR) 

The chemical composition and potential molecular interactions among the latex biomembranes, liposomes, and bioactive compounds (curcumin and papain) were analyzed by Fourier transform infrared (FTIR) spectroscopy using a PRESTIGE 21 spectrometer (Shimadzu, Corporation, Kyoto, Japan). Samples were analyzed in the form of potassium bromide (KBr, Shimadzu) pellets, prepared by mixing approximately 30 mg of KBr with 5% (*w/w*) of sample. We selected this proportion to ensure optimal spectral resolution and transmittance, as higher concentrations can lead to excessive absorbance and signal distortion.

Control spectra of pure curcumin, pure papain, natural latex biomembrane without liposomes, and empty liposomes were recorded for comparative analysis and baseline correction. The pellets were pressed at 80.0 kN 27 for three minutes. For each measurement, 45 scans were recorded with a resolution of 4.0 cm^−1^, covering the spectral range of the region between 4000.00 and 400.0 cm^−1^ in the percentage transmittance mode.

### 4.7. Animals

A total of 45 male Wistar rats, weighing between 240 and 320 g, were used in this study. The research protocol was approved by the Ethics Committee on Animal Experimentation at the University of Brasília (UnB) (under Protocol number 77/2018). The animals were provided by the Catholic University of Brasilia and housed in the vivarium of the Faculty of Health at UnB. The rats were randomly assigned to specific cages (five animals per cage) and provided with water, ad libitum feed and additional feed, such as granola and pumpkin seeds. They were maintained under standard laboratory conditions, with a 12 h light/dark cycle and a constant temperature of 23 °C throughout the study.

#### Experimental Design

**Figure 8:**
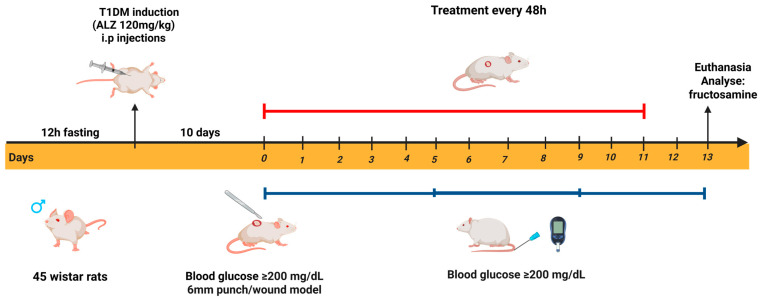
Experimental design of in vivo tests organized into stages. Stage I—Induction of diabetes. Stage II—Wound production. Stage III—Treatment with different biomembranes. Stage IV—Biochemical evaluation. The animals were fasted for 12 h before induction of diabetes. Ten days after induction, diabetes was confirmed, and a surgical wound was created. The animals were then treated with latex-based biomembrane dressings, either without or with bioactive compounds, combined with LED irradiation at a red wavelength (650 ± 20 nm) for 10 min. Treatments were administered every 48 h over 11 days. At the end of the treatment period, the animals were euthanized, and blood and epithelial tissue samples from the dorsal region were collected for further analysis. All experimental procedures were conducted in compliance with the guidelines of the Ethics Committee of the Institute of Biology at UnB. Created in BioRender. Silva, F. (2025) https://BioRender.com/y07m234 (accessed on 16 July 2025).

Figure 8 illustrates the experimental design employed in the in vivo test, which was organized into the following stages: Stage I—diabetes induction; Stage II—lesion production; Stage III—treatment with different biomembranes; and Stage IV—biochemical evaluation.

### 4.8. Diabetic Model Protocol

The animals were fasted for 12 h and then anesthetized intraperitoneally with a solution of ketamine (80 mg/kg) and xylazine (20 mg/kg) (Syntec), adjusted to the weight of each animal. Diabetes was induced by intraperitoneal injection of an alloxan monohydrate solution (120 mg/kg, Sigma-Aldrich Inc., St. Louis, MO, USA) [78,79].

The alloxan solution was prepared by dilution in 0.9% saline solution at a concentration of 60 mg/mL. Due to the photosensitive properties of the compound, the preparation was performed in a dark environment, with the bottle refrigerated and kept away from light until use. Six hours after diabetes induction, a 10% glucose solution was added to the animals’ water bottles and maintained for 24 h to prevent seizures and death due to the hypoglycemic phase.

Ten days after the chemical induction, the animals were subjected to an eight-hour fast, and blood glucose levels were measured. Animals with blood glucose levels ≥ 200 mg/dL were considered diabetic. For blood glucose measurement, the animals were restrained, and their tails were massaged to promote vasodilation of the caudal vein, facilitating blood sampling. A drop of blood was then placed directly onto a glucose test strip. The sampling area was aseptically cleaned with 70% alcohol, and blood was collected using an insulin cannula (13 × 0.45 mm, 26 G). Animals that did not achieve blood glucose levels ≥ 200 mg/dL were subjected to the induction protocol again.

### 4.9. Incisional Wound Model

The diabetic animals were weighed, and their blood glucose levels were measured on days 5, 9, and 13. For the treatment, rats with blood glucose levels exceeding 200 mg/dL had their dorsal hair shaved using a hair clipper (Philips model HC3410/15, Amsterdam, The Netherlands). The animals were then placed in the supine position, and after aseptic preparation, a surgical wound was created using three forceps and a sterile 6 mm punch.

### 4.10. Treatment

The experiment was conducted with 20 animals, as described in Table 2. Each experimental group was designed to retain a final sample size of five animals (*n* = 5). To account for potential losses during the procedures, each group was initially composed of seven animals, providing a buffer of two animals per group to ensure that the minimum required number was maintained throughout the study.

### 4.11. LED Irradiation Procedure

LED phototherapy was performed using a red light source (Rapha device) with a central wavelength of 650 ± 20 nm, chosen for its biostimulatory effects on tissue regeneration. In each session, the device was positioned at a fixed distance of approximately 2 cm above the dorsal wound, with the light beam oriented perpendicularly to the skin surface, covering an irradiated area of 2 cm^2^—matching the size of the applied biomembrane.

This specific wavelength was selected based on prior evidence demonstrating its efficacy in promoting key regenerative processes, such as fibroblast activation, angiogenesis, and collagen synthesis, as well as its favorable tissue penetration profile. Moreover, red light at 650 nm aligns with the absorption peaks of cytochrome c oxidase, a mitochondrial enzyme crucial to photobiomodulation. The selection was also supported by previous clinical studies conducted by our group, in which this wavelength demonstrated a therapeutic benefit when used with natural latex-based biomaterials for treating chronic wounds [80].

The power density (irradiance) was measured using an optical power meter (PM100D; Thorlabs Inc., Newton, New Jersey, USA) positioned at the same distance used in the animal model, yielding a value of 60 mW/cm^2^. The fluence (energy per unit area) was then calculated using the formula below:

Fluence (J/cm^2^) = Power density (W/cm^2^) × Exposure time (s)

Substituting the values,

0.060 W/cm^2^ × 600 s = 36 J/cm^2^

Thus, each 10 min session delivered a fluence of 36 J/cm^2^. The sessions were conducted every 48 h, resulting in a total of six applications over an 11-day treatment period. Measures were taken to minimize the stress and movement of the animals during the procedure, and no signs of thermal damage or phototoxicity were observed. This standardized protocol ensured reproducibility across experimental groups and contributed to the therapeutic effects observed.

### 4.12. In Vivo Evaluation

The animals were monitored for weight loss, water intake, diuresis, and fasting blood glucose levels every two treatment days throughout the experiment. Body weight and blood glucose levels were measured on treatment days, i.e., every 48 h. For this purpose, the animals were placed in a fiber box and weighed using a digital scale (Filizola, São Paulo, Brazil).

### 4.13. Biochemical Analysis (Fructosamine)

At the end of the experimental period, the animals were anesthetized and placed in the supine position. Asepsis was performed in the region where the intracardiac blood puncture was conducted. The collected blood samples were sent to the Veterinary Diagnostic Center in Granja do Torto, Brasília-DF, for biochemical analysis of fructosamine, a marker of average glucose concentration.

### 4.14. Measurement of the Wound Area and Photographic Records

The percentage area of the wound after the postoperative period (induced by the punch injury) was calculated by comparing the changes in the wound size relative to the first day of surgery. Wound healing progression was assessed using ImageJ software (v.1.45) based on images captured during the experiment. The animals were photographed with an iPhone 6s (16 MP camera, Apple Inc., Cupertino, California, USA), and the images were processed with ImageJ software (v.1.45). The percentage of wound contraction was calculated by considering the initial size as 100%, using the following formula [80]:

(1) $$\%\;\mathrm{wound}\;\mathrm{contraction}=\frac{wound\;area\;day\;0\;-\;contracted\;wound\;area\;day(n)}{wound\;area\;day\;0}\times 100$$

A_0_ = wound area in the day 0

A_n_ = wound area in days 3, 5, 7, 9 e 11

### 4.15. Ethics Statement

All animal experiments were conducted in compliance with the guidelines for the care and use of animals approved by the Animal Experimentation Ethics Committee of the UnB. All experimental procedures adhered to the Committee’s regulations. In this study, Wistar rats were used as a model for skin wound healing. The rats were maintained under unrestricted access to standard rodent food and water. Surgical procedures were performed under anesthesia induced by intraperitoneal injection of ketamine–xylazine solution, under fully aseptic conditions. Postoperative pain was managed by subcutaneous administration of ketoprofen (5 mg/kg). Efforts were made to minimize pain and discomfort in the animals throughout the study.

### 4.16. Statistical Analysis

The results of in vivo experiments were analyzed by GraphPad Prisma^®^ 7.0 software, applying specific statistical tests with 95% confidence level (*p* < 0.05). Statistical differences were assessed using one-way ANOVA, followed by post hoc Tukey’s multiple comparisons tests. Additionally, the area under the curve (AUC) of the generated graphical results was calculated to assess the overall effect throughout the experiment, rather than at a single point. These results were subject to one-way ANOVA and Tukey’s comparisons tests, maintaining a 95% confidence level (*p* < 0.05).

## 5. Conclusions

This study demonstrates that LED phototherapy, particularly in the red wavelength range (625–740 nm), significantly enhances the wound healing process when applied in conjunction with natural latex biomembranes (NLBs). The integration of bioactive compounds—curcumin and papain—into the NLB platform, combined with photobiostimulation, resulted in superior wound contraction compared to controls, indicating a synergistic therapeutic interaction between the components.

These findings build upon previous clinical applications of latex-based dressings by adding novel nanocarrier-based functionality. This innovative, cost-effective strategy offers a promising alternative for advancing wound care and improving patient outcomes in both preclinical and clinical contexts.

## 6. Patents

Carneiro, M. L. B., Silva, F. M., Rosa, S. R. F., Santana, T. F., Rosa, S. R. F., Azevedo, R. B., Silva, J. R., and de S. Rodrigues Fleury Rosa, S. (2021). Latex-based biomembranes (Hevea brasiliensis) containing liposome with curcumin (*Curcuma longa*) and papain (*Carica papaya*) and their use associated with LED therapy for the treatment of chronic ulcers and diabetic wounds. Registration number: BR132021001940. INPI—National Institute of Industrial Property. Filed: 2 February 2021.

## Figures and Tables

**Table 1:** Effect of alloxan monohydrate on diabetic rats over a three-week period. Fructosamine levels for each group are expressed as mean ± standard deviation (*n* = 5).

Groups	Fructosamine (µmol/L)
GC (Control)	250 ± 22
G1 (Latex + CUR liposomes + LED)	306 ± 23
G2 (Latex + PAP liposomes + LED)	305.4 ± 22
G3 (Latex + LED)	310.1 ± 25
G4 (Latex + CUR + PAP and + LED)	305.5 ± 22

Abbreviations: CUR—curcumin; PAP—papain; LED—light-emitting diode phototherapy.

**Table 2:** Breakdown of the experimental groups, each containing five animals. Abbreviations: NLB—natural latex biomembrane; CUR—curcumin; PAP—papain; LED—light-emitting diode phototherapy.

Groups	Description	Treatment
CG	Control	No treatment
G1	NLB + CUR liposomes + LED	NLB with curcumin liposomes, associated with LED phototherapy
G2	NLB + PAP liposomes + LED	NLB with papain liposomes, associated with LED phototherapy
G3	NLB + LED	NLB associated with LED phototherapy
G4	NLB + CUR and PAP liposomes + LED	NLB with a combination of curcumin and papain liposomes, associated with LED phototherapy

## Data Availability

The authors confirm that the data supporting the findings of this study are available within the article.
